# Seviteronel, a Novel CYP17 Lyase Inhibitor and Androgen Receptor Antagonist, Radiosensitizes AR-Positive Triple Negative Breast Cancer Cells

**DOI:** 10.3389/fendo.2020.00035

**Published:** 2020-02-11

**Authors:** Anna R. Michmerhuizen, Benjamin Chandler, Eric Olsen, Kari Wilder-Romans, Leah Moubadder, Meilan Liu, Andrea M. Pesch, Amanda Zhang, Cassandra Ritter, S. Tanner Ward, Alyssa Santola, Shyam Nyati, James M. Rae, Daniel Hayes, Felix Y. Feng, Daniel Spratt, Daniel Wahl, Joel Eisner, Lori J. Pierce, Corey Speers

**Affiliations:** ^1^Department of Radiation Oncology, University of Michigan, Ann Arbor, MI, United States; ^2^Program in Cellular and Molecular Biology, University of Michigan, Ann Arbor, MI, United States; ^3^Rogel Cancer Center, University of Michigan, Ann Arbor, MI, United States; ^4^Cancer Biology Program, University of Michigan, Ann Arbor, MI, United States; ^5^Department of Pharmacology, University of Michigan, Ann Arbor, MI, United States; ^6^Division of Hematology and Oncology, Department of Internal Medicine, University of Michigan, Ann Arbor, MI, United States; ^7^Department of Urology, Medicine and Radiation Oncology, University of California, San Francisco, San Francisco, CA, United States; ^8^Innocrin Pharmaceuticals Inc., Durham, NC, United States

**Keywords:** androgen receptor (AR), seviteronel (INO-464), enzalutamide (MDV3100), radiosensitization agents, DNA damage repair (DDR), radiation, TNBC (triple negative breast cancer)

## Abstract

Increased rates of locoregional recurrence (LR) have been observed in triple negative breast cancer (TNBC) despite multimodality therapy, including radiation (RT). Recent data suggest inhibiting the androgen receptor (AR) may be an effective radiosensitizing strategy, and AR is expressed in 15–35% of TNBC tumors. The aim of this study was to determine whether seviteronel (INO-464), a novel CYP17 lyase inhibitor and AR antagonist, is able to radiosensitize AR-positive (AR+) TNBC models. In cell viability assays, seviteronel and enzalutamide exhibited limited effect as a single agent (IC50 > 10 μM). Using clonogenic survival assays, however, AR knockdown and AR inhibition with seviteronel were effective at radiosensitizing cells with radiation enhancement ratios of 1.20–1.89 in models of TNBC with high AR expression. AR-negative (AR−) models, regardless of their estrogen receptor expression, were not radiosensitized with seviteronel treatment at concentrations up to 5 μM. Radiosensitization of AR+ TNBC models was at least partially dependent on impaired dsDNA break repair with significant delays in repair at 6, 16, and 24 h as measured by immunofluorescent staining of γH2AX foci. Similar effects were observed in an *in vivo* AR+ TNBC xenograft model where there was a significant reduction in tumor volume and a delay to tumor doubling and tripling times in mice treated with seviteronel and radiation. Following combination treatment with seviteronel and radiation, increased binding of AR occurred at DNA damage response genes, including genes involved both in homologous recombination and non-homologous end joining. This trend was not observed with combination treatment of enzalutamide and RT, suggesting that seviteronel may have a different mechanism of radiosensitization compared to other AR inhibitors. Enzalutamide and seviteronel treatment also had different effects on AR and AR target genes as measured by immunoblot and qPCR. These results implicate AR as a mediator of radioresistance in AR+ TNBC models and support the use of seviteronel as a radiosensitizing agent in AR+ TNBC.

## Introduction

Each year, it is estimated that over 250,000 women will be diagnosed with invasive breast cancer (1), 15–30% of whom will be diagnosed with triple negative breast cancer (TNBC). This subtype of breast cancer is defined by the lack of estrogen receptor (ER), progesterone receptor, or HER2/*neu* expression and is unresponsive to anti-ER or human epidermal growth factor receptor 2 (HER2) targeting agents. Most patients with TNBC receive multimodal therapy, including surgery, chemotherapy, and radiation therapy (RT), yet TNBC patients still experience the highest rates of locoregional recurrence of any breast cancer subtype. Due to the lack of molecular targeted therapies available for these patients, as well as their intrinsic insensitivity to radiation therapy (2), there is a clinical need for the development of new radiosensitization strategies.

The heterogeneity of TNBC tumors adds to the difficulty of treating this cancer subtype (3, 4). In order to improve response to treatment, it is important to understand the molecular drivers underlying the growth of TNBCs (5). Current molecular therapies for breast cancer patients target the ER or HER2; however, these therapies are ineffective against TNBC due to the lack of ER and HER2 expression (3, 5). Previous studies have established a subgroup of TNBCs which express the androgen receptor (AR) (6), and studies have shown that AR is expressed in 15–35% of all TNBCs (7), rendering AR signaling as a potential target for treatment. Previous work has also suggested an oncogenic role for AR in driving growth of AR-positive (AR+) TNBC (8–10) as well as contributing to invasiveness and migration of TNBC cells (11). Indeed, AR may play multiple roles in breast cancer, both in ER-positive (ER+) and ER-negative tumors, and these results have demonstrated that AR may be an effective target for the clinical treatment of patients with AR+ TNBC (12). Ongoing and completed clinical trials continue to assess the efficacy of AR blockade as a monotherapy for patients with AR+ breast cancers (NCT01889238, NCT01842321, NCT00755885, NCT01808040, NCT01990209, NCT02580448, NCT03383679, NCT02348281, NCT02130700, NCT02067741).

Efforts to target androgen receptor signaling have largely focused on decreasing circulating androgens (CYP17 inhibition) or blocking the binding of androgens to their cognate receptor (AR inhibition) (13–17). Production of androgens is dependent upon the activity of cytochrome P450 17α-hydroxylase/17,20-lyase (CYP17 lyase) (18). Inhibitors of CYP17 lyase have been developed as a strategy for blocking the production of androgens (19). These inhibitors, including the most commonly used CYP17 lyase inhibitor, abiraterone acetate, are used to lower levels of intra-prostatic androgens to treat prostate cancer patients (19–21). Enzalutamide (MDV3100) is a well-characterized second generation anti-androgen which competitively inhibits androgen binding to AR and prevents AR nuclear translocation to block AR binding to DNA (9, 22). In this way, enzalutamide inhibits AR-mediated transcriptional regulation (22). In contrast, seviteronel (INO-464) is a novel inhibitor of both CYP17 lyase and AR. Seviteronel has been shown to be more effective than abiraterone acetate at inhibiting CYP17 lyase (23), and seviteronel also possesses some antagonistic effects against AR, potentially rendering it a dual-AR inhibitor. In phase I studies, seviteronel has been well-tolerated both in men with castration-resistant prostate cancer (CRPC) (24) and in women with ER+ breast cancer or TNBC (25). There is hope that these novel agents, including seviteronel, will be effective in patients with AR+ cancers, including TNBC.

Beyond the role of the androgen receptor in driving cancer cell proliferation, previous work in prostate cancer and breast cancer has demonstrated the role of AR in mediating DNA repair and in the DNA damage response following radiation therapy (26–29). These studies suggest that pharmacologic abrogation of AR both in prostate cancer (darolutamide and enzalutamide) and in AR+ TNBC (enzalutamide) may be a viable treatment strategy for the radiosensitization of aggressive tumors, as AR inhibition may inhibit DNA repair. In addition, following radiation, AR activity increases the expression and phosphorylation of DNAPKcs (26, 28). Therefore, AR inhibition may render cells more sensitive to radiation treatment by reducing activity of DNAPKcs and inhibiting non-homologous end joining (NHEJ), but not homologous recombination (HR) (27). To date, newer generation agents including seviteronel have not been tested as radiosensitizing agents.

Previous work by our group has shown that AR is a mediator of radioresistance in TNBC and that enzalutamide-mediated AR inhibition is sufficient to sensitize AR+ TNBC cells to RT (26). Here we report that seviteronel is able to selectively radiosensitize AR+ TNBC models *in vitro* and *in vivo* and that radiosensitization is mediated, at least in part, through the delayed repair of dsDNA breaks. The mechanism of radiosensitization, however, appears to be different with seviteronel treatment compared to enzalutamide due to differences in AR binding to DNA damage response genes following treatment with seviteronel and radiation.

## Materials and Methods

### Cell Culture

Cell lines were obtained from American Type Culture Collection (ATCC) and grown from frozen samples, including MDA-MB-453 and ACC-422 (AR+ TNBC), MCF-7 (AR−, ER+), and MDA-MB-231 (AR− TNBC). SUM-159 and SUM-185PE (AR+ TNBC) cells were obtained from the University of Michigan stocks generously provided by Dr. Stephen Ethier who isolated the cells while at the University of Michigan. MDA-MB-453 and MDA-MB-231 cells were grown in DMEM (Invitrogen) with 10% fetal bovine serum (FBS, Atlanta Biologicals S11550H). ACC-422 cells were grown in MEM (Gibco) supplemented with 15% FBS (Invitrogen) and 1X Insulin-Transferrin-Selenium-Ethanolamine (ITS-X, Gibco 51500-056). SUM-185 cells were grown in Ham's F-12 media (Gibco) with 5% FBS (Invitrogen), with 0.01M HEPES (Gibco #H3375), 1 μg/mL Hydrocortisone (Sigma #H4001), and 1X ITS-X (Gibco). SUM-159 cells were grown in Ham's F-12 media (Gibco) with 5% FBS (Invitrogen), 0.01M HEPES, 1 μg/mL Hydrocortisone, 1x antibiotic-antimycotic (anti-anti, Thermo Fisher 15240062), and 6 μg/mL insulin (Sigma #I9278). MCF-7 cells were grown in DMEM (Gibco) with 5% FBS (Invitrogen). MDA-MB-453, ACC-422, MCF-7, and MDA-MB-231 cells were supplemented with 1% penicillin/streptomycin (Thermo #15070063). All cell lines were grown in a 5% CO_2_ incubator at 37°C. All cell lines were also authenticated using DNA fingerprinting by short tandem repeat (STR) profiling and routinely tested for mycoplasma (monthly).

### Immunoblotting

Cells were plated in 6-well plates in media containing FBS (MDA-MB-453: 3.5 × 10^5^ cells/well, ACC-422 2.5 × 10^5^ cells/well). The following day, cells were pretreated with charcoal stripped serum (Atlanta Biologics S11650H) for 24 h. Cells were stimulated with 10 nM dihydrotestosterone (DHT, Sigma D-073) and 5 μM enzalutamide (MedChemExpress HY-70002) or seviteronel (Innocrin) and harvested at indicated time points following treatment. Lysis was performed using RIPA buffer (Thermo Fisher 89901) containing protease and phosphatase inhibitors (Sigma-Aldrich PHOSS-RO, CO-RO; Santa Cruz Biotechnology sc-3540, sc-24988A; Cayman Chemical 14333, 14405). Proteins were detected using antibodies for phospho-DNAPKcs (Abcam ab124918, 1:1,000), total DNAPKcs (CST 12311, 1:1,000), androgen receptor (Millipore 06-680, 1:1,000), GAPDH (CST 2118, 1:1,000), and β-Actin (8H10D10, Cell Signaling 12262S, 1:50,000). Secondary antibodies were obtained from Cell Signaling (HRP anti-rabbit CST 7074S, HRP anti-mouse 7076S).

### Viability Assays

Cells were plated in 96 well-plates in media containing FBS and allowed to adhere overnight. The following day the cells were treated with seviteronel or enzalutamide at concentrations ranging from 1 nM to 10 μM. MCF-7 (2,000 cells/well) cells were grown for five days and stained with 0.5% crystal violet. SUM-159 (750 cells/well), MDA-MB-231 (3,500 cells/well), MDA-MB-453 (5,000 cells/well), SUM-185 (4,000 cells/well), and ACC-422 (4,000 cells/well) cells were grown for three days, and cell viability was assessed using Alamar Blue (Thermo Fisher DAL1100). Plates were read on a microplate reader (Cytation 3), and growth was calculated relative to the vehicle control (DMSO). IC50 values for were calculated with GraphPad Prism, and results are reported as the mean ± SEM of three independent experiments. To model hormone deplete conditions, media contained charcoal stripped serum (CSS), without phenol-red were used for MDA-MB-453 and MDA-MB-231 (Gibco 21063-029) and ACC-422 (Gibco 51200-038) cells. Phenol-red free media for ACC-422 cells was supplemented with 2 mM L-glutamine (Sigma G7513). Cells were plated in media containing FBS and were pretreated overnight with CSS before treatment with enzalutamide or seviteronel in concentrations from 1 nM to 10 μM.

### Clonogenic Survival Assays

Cells were plated at single cell density in 6 well-plates and allowed to adhere overnight. Cells were then treated with seviteronel at various doses for 1 h before radiation (2–8 Gy) treatment. Cells were grown for one to four weeks before fixing with methanol/acetic acid and staining with crystal violet. Colonies of 50+ cells were counted and analyzed with the linear quadratic model. Plating densities are outlined in Supplementary Methods.

### γH2AX Immunofluorescence

Cells were plated at a density of 2.5 × 10^5^ cells/well on coverslips in 12 well-plates and allowed to adhere overnight. The following day plates were pretreated with seviteronel (1 and 5 μM) for 1 h before radiation treatment. Cells were fixed at designated time points after radiation using 4% paraformaldehyde. Foci were stained with an anti-phospho-histone H2AX (ser139) antibody (Millipore 05-636), and a fluorescent goat anti-mouse secondary antibody (Invitrogen A11005). Pictures were taken of >100 cells per condition, and γH2AX foci were scored visually by a blinded observer. MDA-MB-453 cells containing ≥ 15 γH2AX foci were scored as positive, and ACC-422 cells with ≥ 10 γH2AX foci were scored as positive. Results were pooled for statistical analysis.

### Xenograft Study

MDA-MB-453 cells were subcutaneously injected bilaterally into the flanks of female CB17-SCID mice. 4.0 × 10^6^ cells were resuspended in 50% Matrigel (BD Biosciences) with PBS. In addition, one 12.5 mg 60 day release DHT pellet (Innovative Research of America) was implanted at the nape of the neck at the time of cell injections. When tumors reached ~80 mm^3^, mice were treated with one of the following treatment conditions: vehicle control (1% CMC, 0.1% Tween-80), seviteronel (75 mg/kg), 2 Gy of RT given once a day for 6 days, or combination of seviteronel with RT with 8 mice in each treatment group. Vehicle control and seviteronel (75 mg/kg) were both administered orally, once daily during treatment. Mice treated with both seviteronel and RT were given seviteronel for 24 h before RT. Tumor growth was measured with digital calipers using the equation: V = L*W2*π/6. Body weight was measured weekly to assess weight loss and toxicity of therapy. All procedures were approved by the Institutional Animal Care and Use Committee (IACUC) at the University of Michigan and comply with regulatory standards.

### Drug Information

DHT was obtained from Sigma (D-073). Enzalutamide was obtained from MedChemExpress (HY-70002). Seviteronel was obtained directly from Innocrin Pharmaceuticals and solubilized in DMSO. Tamoxifen was obtained from MedChemExpress (HY-13757A) and solubilized in DMSO. For cellular assays including clonogenic survival and γH2AX immunofluorescence assays, seviteronel was administered 1 h before radiation treatment.

### ChIP-qPCR

Cells were plated in 10 cm dishes with 4.0 × 10^6^ cells/dish and allowed to adhere overnight before treatment with enzalutamide (1 μM), seviteronel (1 μM), or DMSO control for 18 h before 4 Gy radiation. Cells were harvested 6 h after radiation and crosslinked using formaldehyde (Sigma, F8775). ChIP was performed following the protocol from the HighCell# ChIP kit (Diagenode C01010062) using the anti-androgen receptor antibody (Millipore 06-680). Following immunoprecipitation, DNA was purified using the iPure kit v2 (Diagenode C03010015) and diluted 1:5 for qPCR. qPCR was performed using Fast SYBR Green Master Mix (Thermo Fisher 4385612). Percent of input is reported as the mean ± SEM of two independent experiments. Primers for qPCR are listed in Supplementary Methods (28).

### Reverse Transcription and qPCR

RNA was harvested from cells using QIAzol and extracted using the miRNeasy mini kit (Qiagen 217004). Reverse transcription was performed using SuperScript III Reverse Transcriptase (ThermoFisher 18080085) with random primers (Thermo Fisher 48190011), and cDNA was diluted 1:5. Comparative qPCR was performed using Fast Sybr Green Master Mix (Thermo Fisher 4385612). Plates were read using a QuantStudio6 Flex Real Time qPCR system and analyzed using a comparative method to no treatment control. Relative expression was calculated as compared to gene expression of an untreated control and reported as the mean ± SEM of three independent experiments. Primers for qPCR are listed in Supplementary Methods (26).

### siRNA and Transfections

siRNAs targeting AR were Dharmacon ON-TARGETplus siRNA individual oligos (J-003400-05-0002, J-003400-07-0002). siAR #1: GGAACUCGAUCGUAUCAUU, siAR #2: UCAAGGAACUCGAUCGUAU. Cells were transfected with siRNA using Opti-MEM (Invitrogen 31985-062) and Lipofectamine RNAiMAX (Invitrogen 13778-150). For clonogenic survival assays, cells were transfected with siRNA and irradiated, then plated at cell densities as outlined in Supplementary Methods.

### Radiation

Irradiation of cells and mice was performed at the University of Michigan Experimental Irradiation Core using a Philips RT250 (Kimtron Medical). In keeping with previous studies, the dose rate was ~2 Gy/min as previously described (30, 31).

### Statistical Analyses

Statistical tests were performed in GraphPad Prism 7.0. Statistics for *in vitro* experiments were performed using a two-sided Student's *t*-test to compare gene expression between cells treated with DMSO and enzalutamide. For immunoblot comparisons, a one-way ANOVA with Dunnett's multiple comparisons test was used. For *in vivo* studies, a one-way ANOVA was used to compare tumor volume, and a log-rank test was used to compare survival curves. Synergy between seviteronel with radiation was assessed using the fractional tumor volume (FTV) method for *in vivo* experiments as previously described (32, 33).

## Results

### AR Inhibition Alone Does Not Affect Viability of AR+ TNBC Cells

**Figure 1:**
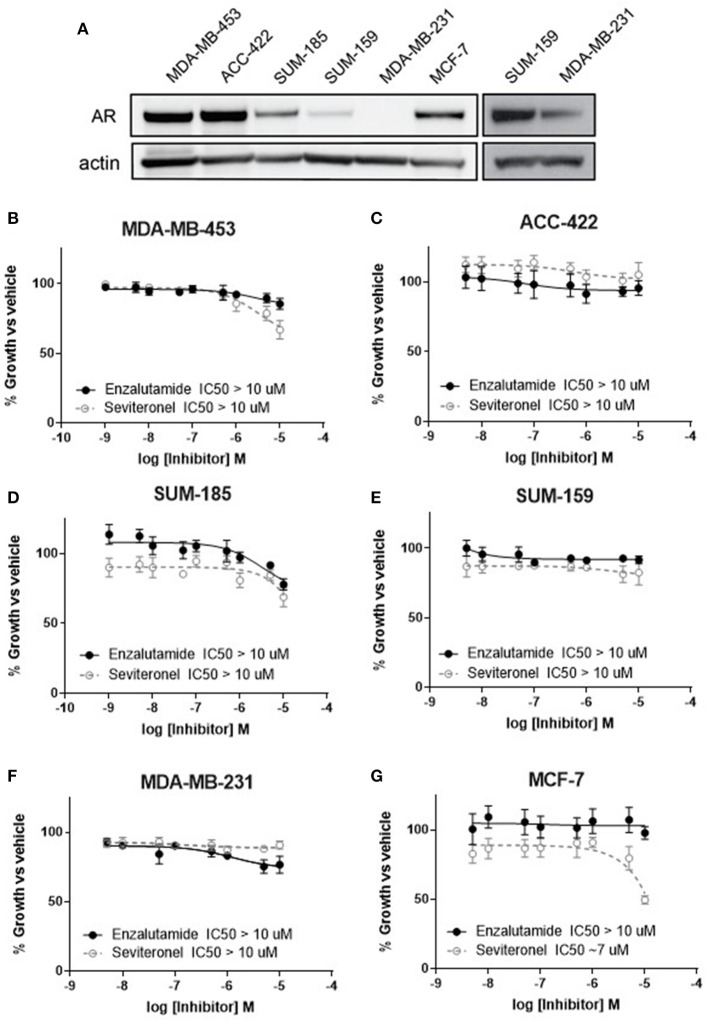
Cell viability is not affected by AR inhibition with enzalutamide or seviteronel. **(A)** AR expression was measured via immunoblot in TNBC and ER+ cell lines. Cells were treated with seviteronel or enzalutamide, and viability was assessed via metabolic activity for AR+ TNBC cells: **(B)** MDA-MB-453, **(C)** ACC-422, **(D)** SUM-185, **(E)** SUM-159, and **(F)** AR− TNBC MDA-MB-231 cells. Viability of AR−, ER+ MCF-7 cells was assessed with **(G)** enzalutamide and seviteronel treatment at concentrations up to 10 μM. Graphs represent mean ± SEM for three independent experiments.

Anti-androgen therapies have been effective at inhibiting the growth of AR+ prostate cancer cells due to their reliance on AR signaling. Similarly, one strategy for inhibiting the growth of AR+ TNBC cell line models has been the use of AR inhibitors as monotherapy (34, 35). Here we compared two AR-antagonists, seviteronel and enzalutamide, in their ability to inhibit viability of TNBC cells *in vitro*. Levels of AR protein were initially assessed in five TNBC cell lines and one ER+ cell line (Figure 1A). Triple-negative breast cancer cell lines with the highest AR expression were selected for subsequent study, including MDA-MB-453, ACC-422, and SUM-185^28^. In AR+ TNBC cells lines, MDA-MB-453, ACC-422, SUM-185, and SUM-159, treatment with enzalutamide or seviteronel did not cause a significant decrease in viability at concentrations up to 10 μM (Figures 1B–E). Similar results were observed when ACC-422 and MDA-MB-453 cells were treated with enzalutamide or seviteronel in media containing CSS and lacking phenol red (Supplementary Figures 1A,B). In an AR− TNBC model, MDA-MB-231 cells, treatment with seviteronel or enzalutamide did not decrease cell viability at concentrations up to 10 μM when grown in FBS (Figure 1F) or CSS (Supplementary Figure 1C).

Seviteronel has also been reported to have some anti-estrogen activity, so similar experiments were performed in an ER+ cell line with low AR expression (MCF-7 cells). While the selective estrogen receptor modulator tamoxifen is able to inhibit viability of ER+ MCF-7 cells in a dose-dependent manner (IC50 = 1.1 μM, Supplementary Figure 1D), treatment with enzalutamide did not significantly inhibit viability (IC50 > 10 μM, Figure 1G). Seviteronel, however, also had some antagonistic effects on MCF-7 cells with an IC50 ~7 μM. This may be due to the anti-estrogenic effects of seviteronel in reducing CYP17 lyase activity, which has been previously reported (25). These results suggest that AR inhibition does not affect cell viability at concentrations up to 10 μM in AR+ TNBC cell lines, and inhibition of AR alone at these concentrations may not be sufficient to inhibit viability of AR+ TNBC cells *in vitro*.

### AR Knockdown or Seviteronel Treatment Radiosensitizes AR+ TNBC Cells *in vitro*

**Figure 2:**
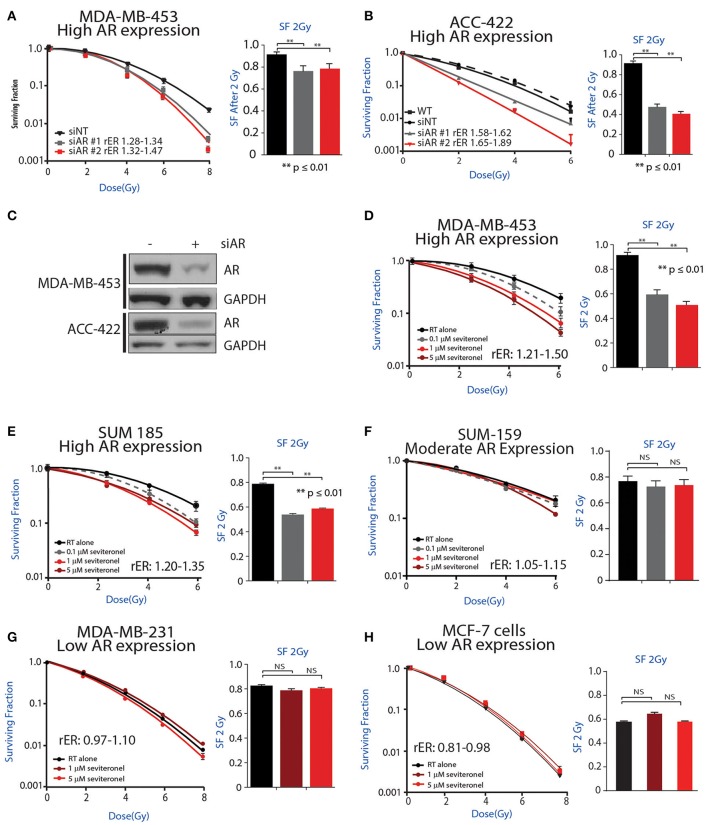
AR inhibition via genetic knockdown or seviteronel treatment in combination with radiation decreases clonogenic survival in AR+ TNBC cell lines. Clonogenic survival assays with multiple siRNAs targeting AR were performed in **(A)** MDA-MB-453 and **(B)** ACC-422 cells. **(C)** Knockdown was confirmed by immunoblot. Clonogenic survival assays with seviteronel were performed in TNBC cell lines with high AR expression including **(D)** MDA-MB-453 and **(E)** SUM-185 cells, and a moderate AR-expressing cell line **(F)** SUM-159. Assays were also performed in AR− TNBC **(G)** MDA-MB-231 cells or AR−, ER+ **(H)** MCF-7 cells. Representative clonogenic survival assays for each cell line are shown, while the surviving fractions of cells at 2Gy (SF 2Gy) are shown as the mean ± SEM of three independent experiments. NS, *p* is not significant, **p ≤ 0.01.

Initially, to determine whether AR knockdown is sufficient to confer radiosensitivity in AR+ TNBC cells, clonogenic survival assays were performed with siRNA-mediated knockdown of AR in multiple AR+ TNBC cell lines. Knockdown of AR with multiple siRNAs was performed in MDA-MB-453 cells, resulting in increased radiosensitivity with radiation enhancement ratios (rER) of 1.28–1.47. There was also a significant decrease in surviving fraction of cells at 2 Gy (Figure 2A). Comparatively, cisplatin, a well-characterized radiosensitizer, provides enhancement ratios of 1.2 (36, 37). Similar to results observed in MDA-MB-453 cells, a significant decrease in the surviving fraction at 2 Gy was observed in ACC-422 cells with siRNA-mediated AR knockdown (Figure 2B) with enhancement ratios of 1.58–1.89. siRNA knockdown of AR was verified by western blot in MDA-MB-453 and ACC-422 cells (Figure 2C).

To further address how AR is involved in the radiation response, radiosensitization was assessed via clonogenic survival assays with seviteronel-mediated AR inhibition in multiple AR+ models of TNBC. Doses of seviteronel were selected to be 10–100 fold lower than the IC50 of the drug to evaluate radiosensitizing effects independent of cytostatic or cytotoxic effects of seviteronel as a single agent. In AR+ TNBC cell lines, treatment with seviteronel provided a dose-dependent increase in radiosensitivity. In MDA-MB-453 cells, treatment with seviteronel led to significant radiosensitization with radiation enhancement ratios from 1.21 to 1.50 and a significant decrease in the surviving fraction of cells at 2 Gy (Figure 2D). Similarly, in SUM-185 and SUM-159 cells, the radiation enhancement ratios with seviteronel were 1.20–1.35 and 1.05–1.15, respectively (Figures 2E,F). SUM-185 cells also had a significant decrease in the surviving fraction of cells after 2 Gy radiation suggesting that seviteronel-mediated AR inhibition is effective at sensitizing TNBC cells that have high AR expression, even with low doses of RT (38).

In contrast, in MDA-MB-231 cells, a TNBC model with low AR expression, seviteronel treatment did not result in significant radiosensitization (rER = 0.97–1.10) or a significant decrease in the surviving fraction of cells at 2 Gy (Figure 2G). Similar results were seen in MCF-7 cells (Figure 2H) which have high ER expression, but low AR expression (rER = 0.81–0.98). Together, these results suggest that seviteronel-mediated AR inhibition is able to radiosensitize AR+ TNBC models *in vitro*. In breast cancer cell lines that lack AR, however, treatment with seviteronel does not lead to radiosensitization (Figure 2G), suggesting the effect is mediated through the androgen receptor.

### Differential Effects on AR Targets With Enzalutamide- or Seviteronel-Mediated Inhibition

**Figure 3:**
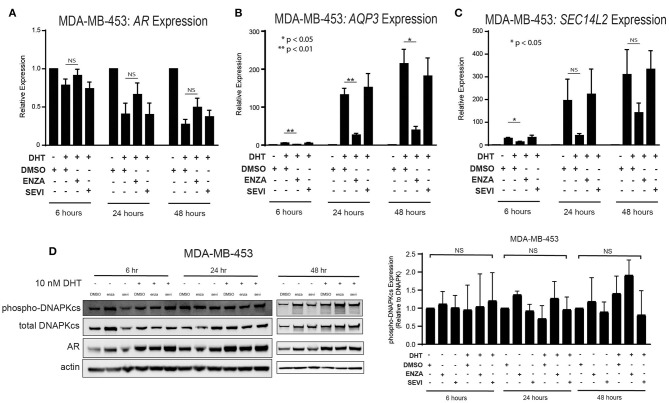
Differential effects on AR and AR targets with enzalutamide and seviteronel treatment. AR+ TNBC cells were treated with 5 μM enzalutamide or seviteronel ± 10 nM DHT. RT-qPCR was used to assess mRNA expression of **(A)** *AR*, **(B)** *AQP3*, and **(C)** *SEC14L2* in MDA-MB-453 cells. **(D)** Protein levels of p-DNAPKcs, total DNAPKcs, and AR were measured by immunoblot in MDA-MB-453 cells. Gene expression data represent mean ± SEM for three independent experiments, and immunoblots are representative of triplicate experiments. NS, *p* is not significant, *p < 0.05, **p < 0.01.

The role of the androgen receptor to signal as a transcription factor has been well-characterized in prostate cancer and is increasingly being recognized and studied in breast cancer. AR nuclear translocation results in the activation of downstream target genes including *AQP3* and *SEC14L2* (39). Activation of target genes was studied in two TNBC cell lines with high AR expression: MDA-MB-453 and ACC-422 cells. Following stimulation with DHT, there is a decrease in AR transcript expression in MDA-MB-453 cells (Figure 3A). Treatment with enzalutamide and DHT, however, results in increased levels of AR mRNA in comparison to control cells also stimulated with DHT. Following DHT stimulation, AR inhibition with enzalutamide also decreases mRNA levels of target genes (*AQP3, SEC14L2*) in AR+ TNBC (Figures 3B,C). Seviteronel-mediated inhibition, however, results in no significant change in expression of AR target genes. Similar results were also seen in ACC-422 cells following DHT stimulation and treatment with enzalutamide or seviteronel (Supplementary Figures 2A–C). In addition, AR activation has been shown to increase levels of phosphorylation of the catalytic subunit of DNA protein kinase (p-DNAPKcs) following radiation (26, 28). Treatment with only enzalutamide or seviteronel following DHT stimulation in the absence of radiation does not result in changes in p-DNAPKcs levels, total levels of DNAPKcs, or AR protein in MDA-MB-453 (Figure 3D, Supplementary Figures 3A,B) or ACC-422 cells (Supplementary Figures 2D–G). Together these results indicate differences between enzalutamide- and seviteronel-mediated AR inhibition and the effects on downstream AR target genes.

### AR Inhibition Results in Persistence of dsDNA Breaks After Radiation

**Figure 4:**
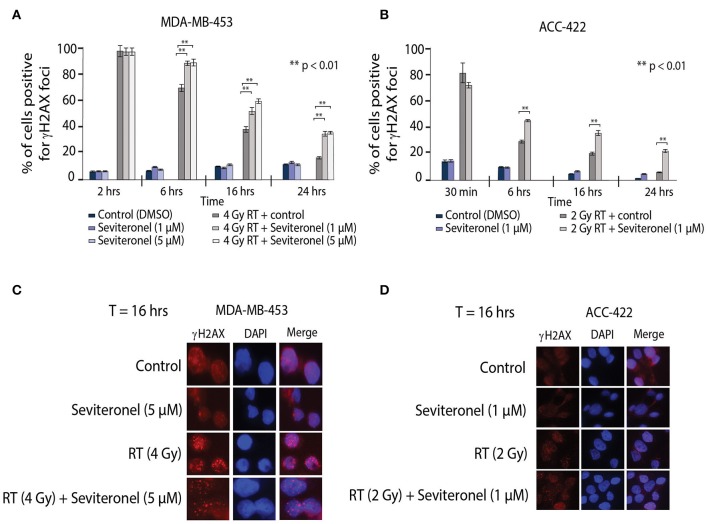
Combination treatment results in increased levels of γH2AX foci and delayed resolution of dsDNA breaks. γH2AX foci in **(A)** MDA-MB-453 (p53 wild type) and **(B)** ACC-422 (p53 mutant) cells were observed using a fluorescent microscope. MDA-MB-453 cells containing ≥15 foci were counted as positive, and ACC-422 cells containing ≥10 foci were counted as positive. Representative images of γH2AX foci are shown for **(C)** MDA-MB-453 and **(D)** ACC-422 cells for each treatment group. Graphs represent the mean ± SD for three independent experiments (**p < 0.01).

Ionizing radiation induces single and double strand breaks in DNA that are acted upon by distinct DNA repair pathways. If unrepaired, single strand DNA breaks can be converted into dsDNA breaks at stalled replication forks; dsDNA breaks then require repair through NHEJ or HR repair pathways. Therefore, to further understand how seviteronel mediates radiosensitization *in vitro*, cells were stained for γH2AX foci to assess levels of dsDNA breaks following RT in multiple models of AR+ TNBC. When AR+ TNBC cells (MDA-MB-453 and ACC-422) were treated with seviteronel alone, there was no change in levels of γH2AX positive cells at 2, 6, 16, or 24 h in MDA-MB-453 cells (Figure 4A) or at 30 min, 6, 16, or 24 h in ACC-422 cells (Figure 4B), suggesting that seviteronel alone does not induce widespread dsDNA breaks. As expected, RT alone (2–4 Gy) rapidly induces dsDNA breaks that were slowly resolved over ~16–24 h, depending on the cell line used. Differences in the p53 status of MDA-MB-453 (wild type) and ACC-422 (mutant) (6) cells, however, do not appear to affect dsDNA break repair (40). Combination treatment with radiation and seviteronel at 1 or 5 μM led to significant delays in dsDNA break repair in both cell lines as indicated by significantly higher levels of γH2AX positive cells compared to cells treated with radiation alone at the same time points. Representative images of cells at 16 h after RT are shown for both cell lines (Figures 4C,D). These results suggest that seviteronel-mediated AR inhibition results in accumulation of dsDNA breaks following radiation in AR+ TNBC models, including MDA-MB-453 and ACC-422 cell lines.

### Seviteronel Treatment in Combination With Radiation Delays Growth of Xenograft Tumors

**Figure 5:**
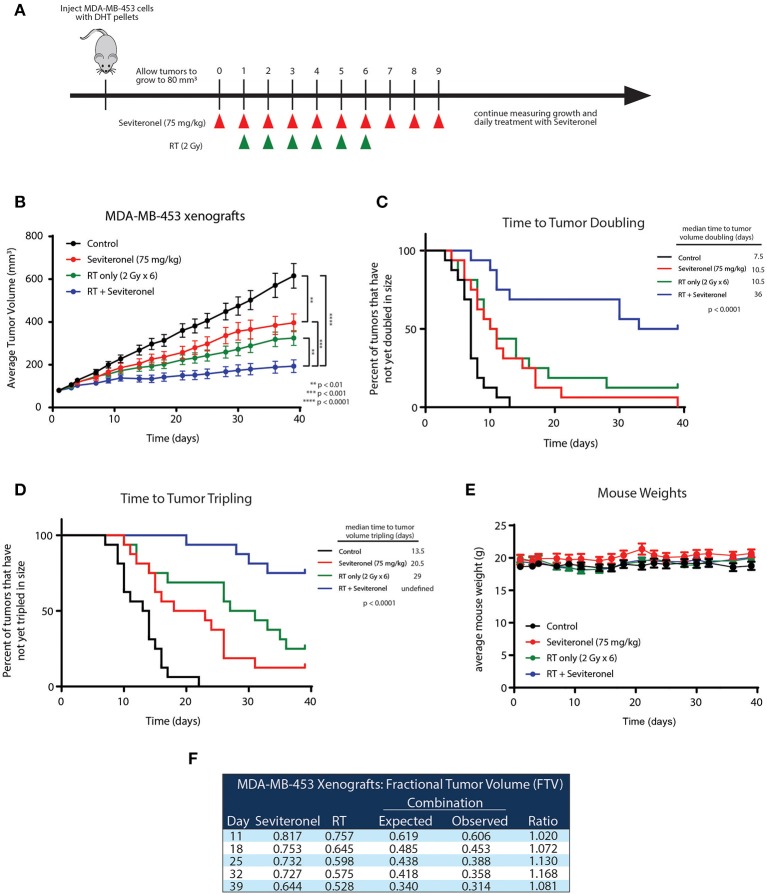
Seviteronel with radiation is more effective than seviteronel or radiation alone in MDA-MB-453 xenograft model *in vivo*. **(A)** MDA-MB-453 cells were injected into CB17-SCID mice, and treatment began when tumors reached ~80 mm^3^ in size. Treatment with seviteronel began one day prior to initiation of radiation treatment and continued after the completion of six fractions of radiation. **(B)** Tumor volume was measured, **(C)** time to tumor doubling, and **(D)** time to tumor tripling was assessed. **(E)** Toxicities were evaluated by animal weights throughout the duration of treatment and monitoring. **(F)** The FTV method was used to assess synergy of combination treatment of seviteronel with radiation (**p < 0.01, ***p < 0.001, ****p < 0.0001).

To further validate the *in vitro* radiosensitization findings and determine whether these occurred in intact tumors, an *in vivo* xenograft model with MDA-MB-453 cells was used. MDA-MB-453 cells were injected subcutaneously into bilateral flanks of CB17-SCID mice. When tumors reached ~80 mm^3^, seviteronel was administered orally each day. In order to assess true radiosensitization, seviteronel treatment was started one day prior to the beginning of radiation to achieve plasma concentrations in the 5 μM range at time of first radiation treatment (Figure 5A). In contrast to the *in vitro* viability assays, xenograft tumor growth was significantly inhibited by seviteronel alone and, as expected, was also inhibited by RT alone. The combination treatment with seviteronel and RT, however, led to a much more significant decrease in tumor volume compared to either treatment alone (Figure 5B). In addition, there was a significant delay in time to tumor doubling (7.5 vs. 36 days) and tripling (13.5 days vs. undefined) in the mice treated with seviteronel and radiation compared to control mice (Figures 5C,D). The combination treatment seemed to be well-tolerated, as there were no differences in weights or activity levels of the mice (Figure 5E). Using the fractional tumor volume (FTV) method for assessing synergy, the combination of seviteronel with radiation was found to have a synergistic effect (not just additive) with ratios >1 (Figure 5F). Together these results suggest that seviteronel treatment in combination with radiation is effective at slowing tumor growth *in vivo* and that the combination treatment was more effective than either therapy alone.

### AR Binding at DNA Damage Response Genes Is Enhanced With RT and Seviteronel-Mediated AR Inhibition

Having demonstrated that seviteronel-mediated AR inhibition is sufficient to confer radiosensitization in AR+ models of TNBC and that dsDNA breaks persist longer with combination treatment than with RT alone, we sought to better understand the mechanism by which seviteronel mediates radiosensitization. We hypothesized that AR transcriptional activity was regulating DNA damage gene expression to influence DNA repair. Therefore, inhibition of AR with seviteronel or enzalutamide would decrease target gene expression and AR binding to AR-transcription factor binding sites located near or within DNA repair genes. Using ChIP-qPCR, we evaluated AR recruitment at DNA damage response genes containing AR binding regions in an effort to understand how seviteronel was influencing the DNA damage response following radiation compared to AR inhibition with enzalutamide. Previous work from our lab suggests that that AR may be important in AR+ TNBC for the repair of dsDNA breaks by activating DNAPKcs (26), an important protein involved in NHEJ (41). A number of DNA damage response genes have previously been reported to be controlled by AR expression in prostate cancer models, including *XRCC2, XRCC3*, and *PRKDC* (28). *XRCC2* and *XRCC3* contain AR regulatory regions, and these genes are part of the Rad51 family, playing an important role in the repair of dsDNA breaks through HR (42). *PRKDC* is the gene encoding DNAPKcs. At all three loci, AR binding is thought to influence gene expression.

**Figure 6:**
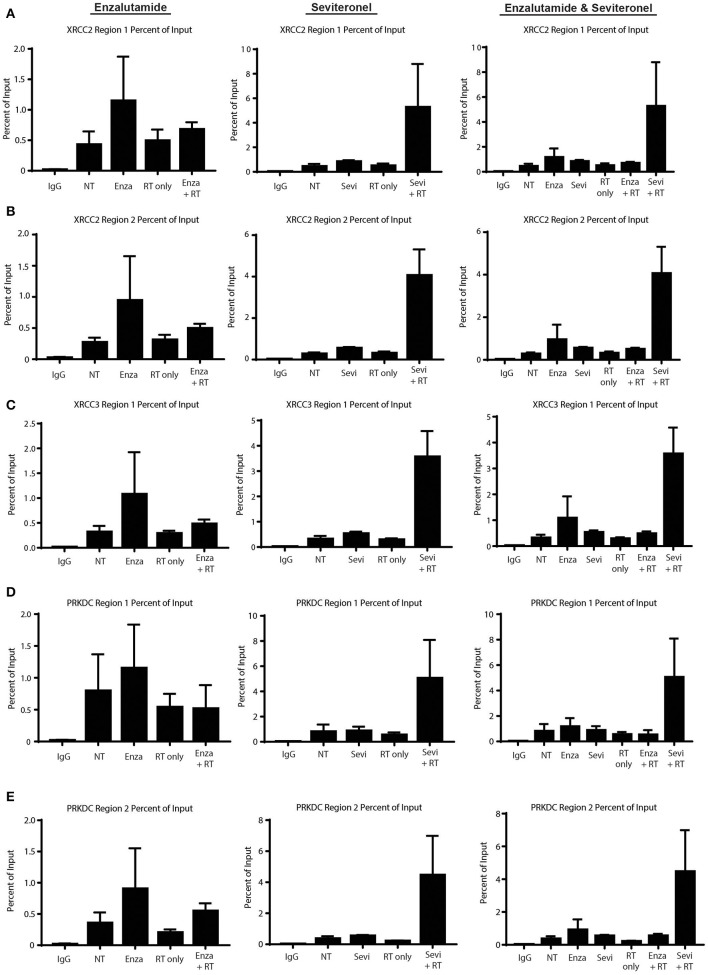
Seviteronel with radiation increases AR recruitment compared to monotherapy treatment of enzalutamide with radiation. AR recruitment to DNA damage response genes was measured by ChIP-qPCR experiments at AR binding to regions surrounding **(A,B)** *XRCC2*, **(C)** *XRCC3*, and **(D,E)** *PRKDC*. Graphs represent the mean ± SEM for two independent experiments. NT, no treatment/control.

To begin to understand how enzalutamide and seviteronel may be differentially affecting expression of AR-controlled genes following radiation, ChIP-qPCR experiments were performed. AR recruitment to AR-occupied regions was compared between the following treatment conditions: control (no treatment), enzalutamide only, seviteronel only, radiation alone, or the combination of enzalutamide and radiation, or seviteronel and radiation. When cells were treated with seviteronel or enzalutamide alone, AR was recruited to regulatory regions of *XRCC2, XRCC3*, and *PRKDC* (Figures 6A–E). When compared to cells treated with radiation alone, cells receiving both radiation and seviteronel had significantly increased AR recruitment. Similar AR binding was not observed when cells were treated with combination of enzalutamide and radiation, suggesting that this is a seviteronel-specific effect.

## Discussion

Here we show that although seviteronel and enzalutamide exhibited limited effect as a single agent (IC50 > 10 μM), AR knockdown and AR inhibition with seviteronel were effective at radiosensitizing AR+ TNBC models with radiation enhancement ratios of 1.20–1.89. Radiosensitization of AR+ TNBC models was at least partially dependent on impaired dsDNA break repair. Similar effects were observed *in vivo* where there was a significant reduction in tumor volume and a delay to tumor doubling and tripling times in mice with AR+ TNBC xenograft tumors treated with seviteronel and radiation. Mechanistically, we report differential binding of AR to target genes in the presence of enzalutamide and seviteronel, suggesting different mechanisms of action between the two drugs.

These findings should be taken in the broader context of anti-androgens as a therapeutic strategy in breast cancer. Other groups have investigated how AR inhibition may be a therapeutic strategy for aggressive TNBC tumors. Clinical trials with enzalutamide as monotherapy have demonstrated that AR inhibition is safe and efficacious (43), and patients with AR-activated tumors who receive enzalutamide have improved metastatic progression-free survival (44). Additional studies are investigating the use of CYP17 lyase inhibitors, like abiraterone acetate, which may be effective for patients with molecular apocrine tumors (45). Similarly, a trial investigating treatment with seviteronel for patients with breast cancer (NCT02580448) was recently completed, and stage 1 results from the Phase II trial suggest that seviteronel provides clinical benefit and decreased levels of circulating tumor cells when administered alone (25, 46). This work demonstrates additional clinical applications for AR targeting agents in the treatment of breast cancer.

There are also a number of limitations of the current study. While this study suggests that AR inhibition is an effective strategy for the radiosensitization of AR+ TNBC cells, additional studies are needed to understand the exact mechanism of radiosensitization in these models, and confirmation using additional AR+ TNBC models, including patient derived xenograft (PDX) models are still needed. Future work will also seek to understand the differences in how enzalutamide and seviteronel affect the ability of AR to bind DNA and activate the transcription of downstream target genes. Our results suggest that seviteronel has a unique mechanism of radiosensitization compared to the second generation anti-androgen enzalutamide. Indeed, these results suggest that AR is increasingly recruited to binding sites of DNA damage response genes involved both in HR and NHEJ following treatment with seviteronel and radiation. This may suggest that AR remains bound to these regions but may not be activating transcription of these genes. This may be due to co-repressor recruitment at these sites (instead of co-activator) or stalling of the transcriptional machinery. Thus, although seviteronel is found more frequently bound to promoter regions of NHEJ and HR genes, there does not seem to be a functional improvement of DNA repair efficacy or efficiency, suggesting that the mechanism of radiosensitization with seviteronel is different than that previously reported for enzalutamide. Although the details of these mechanistic differences remain unresolved, additional studies are underway to investigate the mechanism of AR-mediated radiosensitization both with enzalutamide and seviteronel to understand how these AR inhibitors are differentially affecting the radiation response. Another limitation is the disparate findings on the effect of seviteronel *in vitro* and *in vivo*. Indeed, this study demonstrates that although AR inhibition with seviteronel alone is not sufficient to inhibit the viability of AR+ TNBC cells *in vitro*, seviteronel does inhibit proliferation *in vivo* and sensitizes cells to radiation treatment both *in vitro* and *in vivo* (Figures 1, 2, 5). This difference may be attributed to a difference in intratumoral androgens, which would be inhibited *in vivo* but not affected in tissue culture, *ex vivo* studies. Furthermore, cytostatic effects of a drug tend to have a more significant impact *in vivo* compared to the *in vitro* cell proliferation studies performed, as these are compared to vehicle controls. Finally, seviteronel may have cancer cell extrinsic effects, including altering the tumor microenvironment and endocrine signaling within the mice that would not be observed to the same extent *in vitro*.

In summary, TNBC continues to be a clinically challenging disease entity with limited/no effective molecularly targeted therapies. With the identification of AR+ TNBC subtype, interest in targeting AR in these patients continues. The data reported herein provide the preclinical rationale for continued clinical investigation of anti-androgens as a general class of molecularly targeted therapies for the targeted treatment of AR+ TNBC and specifically for the further investigation of seviteronel as a radiosensitizing agent in women with radioresistant AR+ TNBC.

## Data Availability Statement

The datasets generated for this study are available on request to the corresponding author.

## Ethics Statement

The animal study was reviewed and approved by Institutional Animal Care and Use Committee (IACUC) at the University of Michigan.

## Author Contributions

AM, BC, EO, KW-R, LM, ML, AP, AZ, CR, SW, AS, and SN conducted experiments and analyzed the data. All authors contributed to manuscript idea and revision, read and approved the submitted version.

### Conflict of Interest

JE was previously employed by Innocrin Pharmaceuticals Inc. JE is no longer employed by Innocrin. The remaining authors declare that the research was conducted in the absence of any commercial or financial relationships that could be construed as a potential conflict of interest.
