# Supplementary material for: Seviteronel, a Novel CYP17 Lyase Inhibitor and Androgen Receptor Antagonist, Radiosensitizes AR-Positive Triple Negative Breast Cancer Cells
Source: Front Endocrinol (Lausanne). 2020 Feb 11;11:35. doi: 10.3389/fendo.2020.00035 (PMC7027396; doi:10.3389/fendo.2020.00035)
Supplement: Supplementary file 1 [file Table_1.docx]

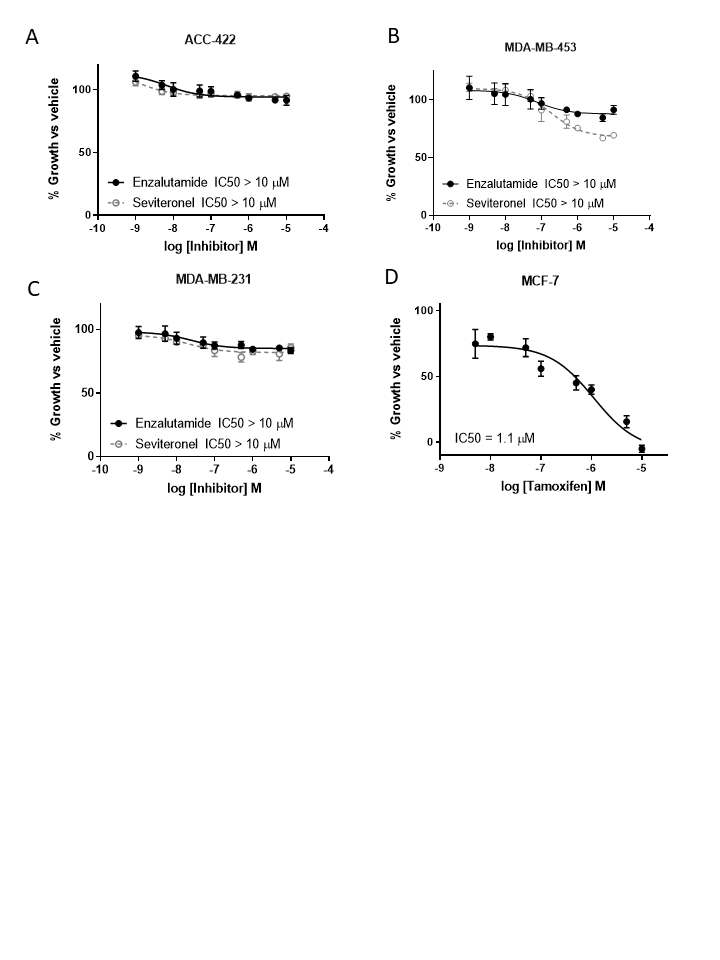


**Supplementary Figure 1: Cell viability with inhibitor treatment**

AR+ TNBC cell lines were pretreated with CSS and phenol free media overnight before treatment with enzalutamide or seviteronel. Cell viability was assessed via metabolic activity in (**A**) ACC-422 and (**B**) MDA-MB-453 cells. (**C**) Viability of MDA-MB-231 cells, an AR- TNBC cell line, was used as a control. (**D**) Viability of MCF-7 cells was inhibited with treatment of tamoxifen with an IC50 of 1.1 μM. Graphs represent mean ± SEM for triplicate experiments.


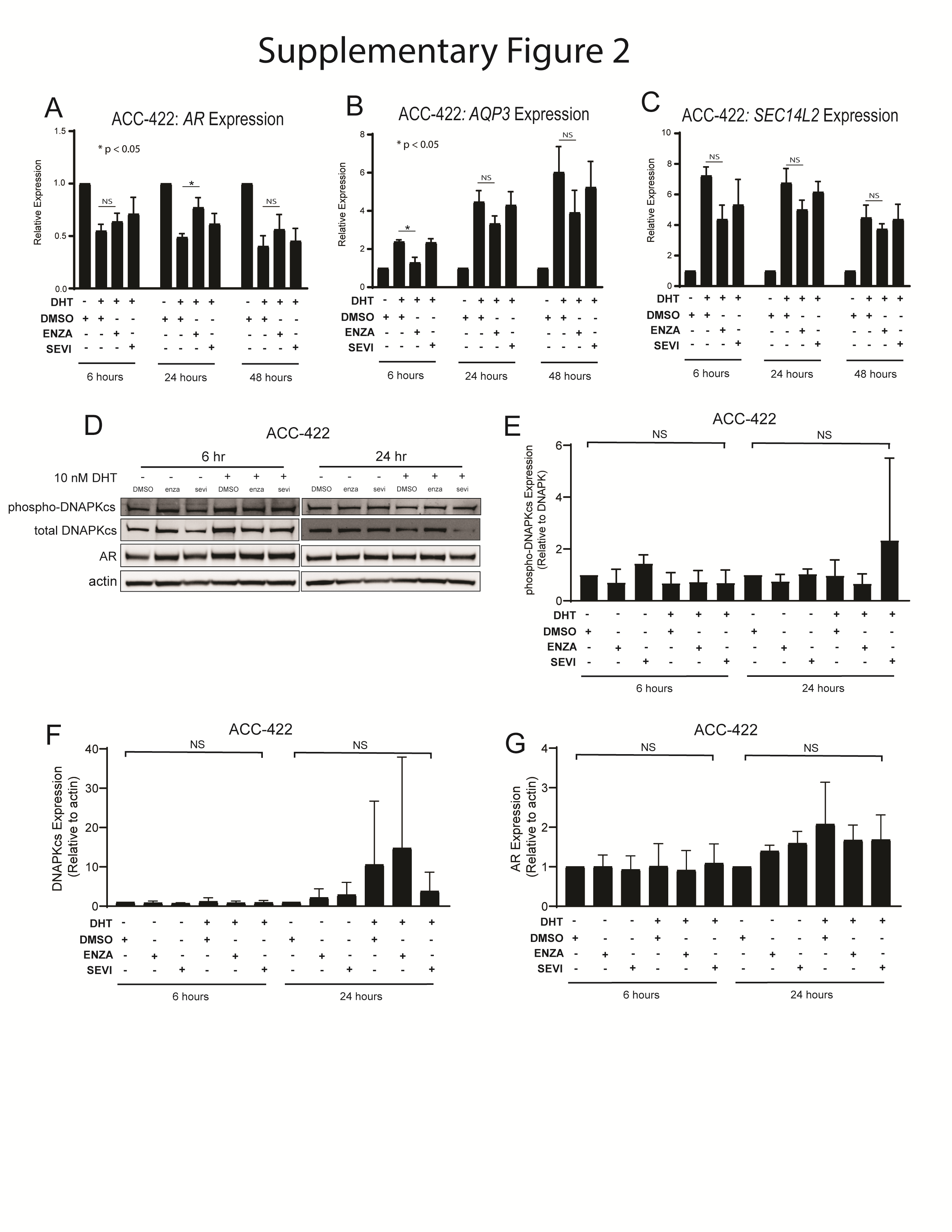


**Supplementary Figure 2: Differential effects on AR and AR targets with enzalutamide and seviteronel in ACC-422 cells.**

ACC-422, AR+ TNBC cells, were treated with 5 µM enzalutamide or seviteronel ± 10 nM DHT. mRNA expression was assessed via qPCR for (**A**) *AR*, (**B**) *AQP3*, (**C**) *SEC14L2*. Expression of p-DNAPKcs, total DNAPKcs, and AR protein levels were measured by immunoblot (**D**) and quantified (**E-G**). Gene expression data represent three independent experiments and are shown as mean ± SEM. Immunoblots are representative of triplicate experiments. (NS = p is not significant, * = p < 0.05).


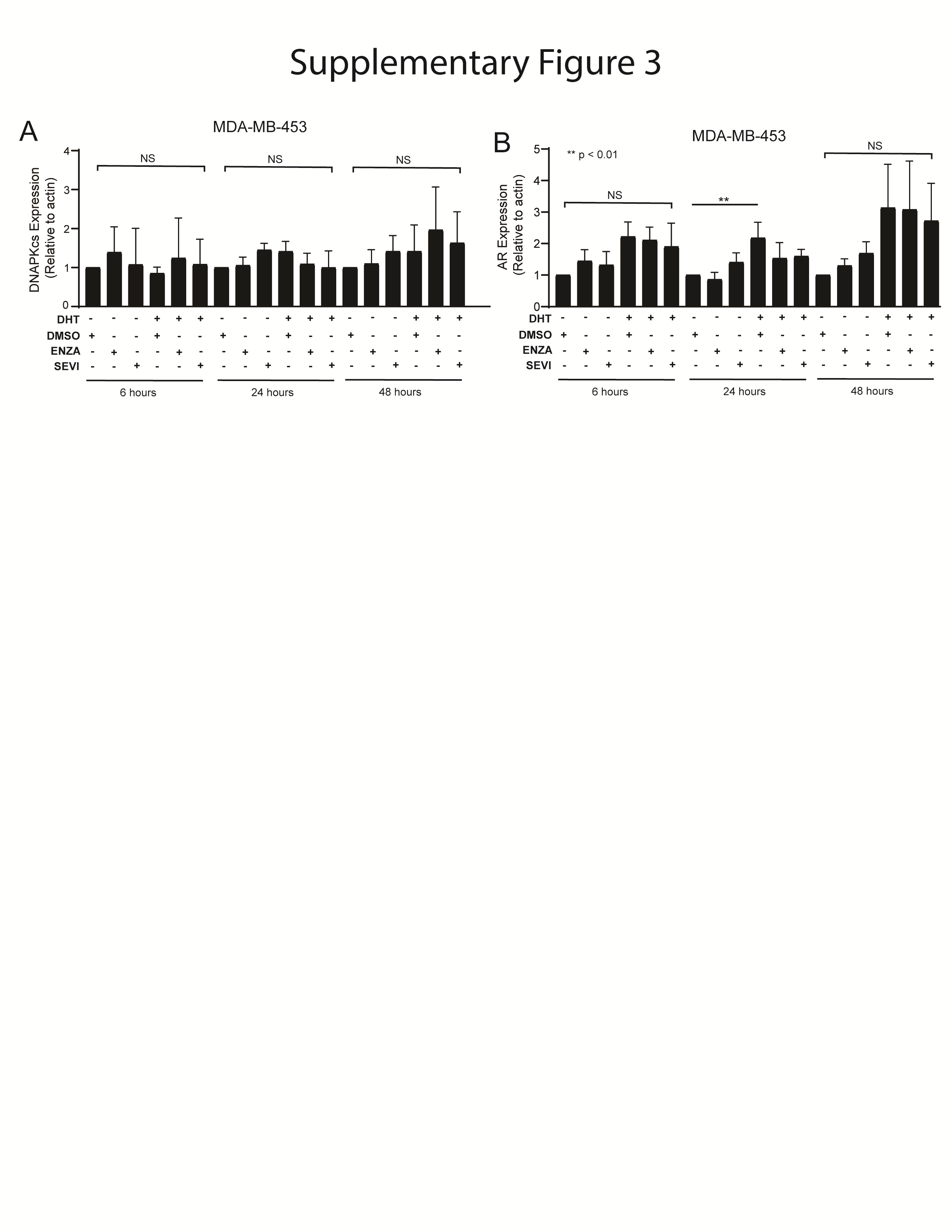


**Supplementary Figure 3: Quantification of DNAPKcs and AR Protein Expression in MDA-MB-453 cells**

Quantification of immunoblots for (**A**) DNAPKcs and (**B**) AR expression in MDA-MB-453 cells. (NS = p is not significant, ** = p < 0.01).

**Supplementary Methods:**

**Table 1: qPCR primers**

| XRCC2_AROR1_F1 | GCCTGAACAATGGAGATAAAAGAG |
| --- | --- |
| XRCC2_AROR1_R1 | TGCCTCAGGGAACAAATAAGAC |
| XRCC2_AROR2_F1 | AGCCAAAACACTCCCTCAAG |
| XRCC2_AROR2_R1 | CTCAAGTCATCTTCCCACCTC |
| XRCC3_AROR1_F1 | GCCAGCGTTTTGTTAACCTG |
| XRCC3_AROR1_R1 | GGATTTGGATCTACTGGACCTG |
| PKRDC_AROR1_F1 | GCATCGCTAGGGAACAAGG |
| PKRDC_AROR1_R1 | CTGCGATAAACATCTTGACAGAG |
| PKRDC_AROR2_F1 | AAGGTGTCACTTCCTGTTCAC |
| PKRDC_AROR2_R1 | TGAGCTATGCTGATTTTACCTAGG |
| AR Forward | CAGTGGATGGGCTGAAAAAT |
| AR Reverse | GGAGCTTGGTGAGCTGGTAG |
| AQP3 Forward | CCGTGACCTTTGCCATGTGCTT |
| AQP3 Reverse | TTGTCGGCGAAGTGCCAGATTG |
| SEC14L2 Forward | CCTGAAGACCAAGATGGGAGAG |
| SEC14L2 Reverse | GCTGTAGGTGTTGTCAAACCGC |

**Table 2: Plating Densities for Clonogenic Survival Assays**

|  | **MDA-MB-453** | **ACC-422** | **SUM-185** | **SUM-159** | **MDA-MB-231** | **MCF-7** |
| --- | --- | --- | --- | --- | --- | --- |
| **0 Gy** | 250 | 500 | 300 | 150 | 250 | 500 |
|  | 500 | 1000 |  |  |  |  |
| **2 Gy** | 1000 | 1000 | 600 | 300 | 500 | 1000 |
|  | 2000 | 2000 |  |  |  |  |
| **4 Gy** | 2000 | 10,000 | 1200 | 300 | 1000 | 5000 |
|  | 4000 | 20,000 |  |  |  |  |
| **6 Gy** | 2500 | 20,000 | 4500 | 450 | 2500 | 10,000 |
|  | 5000 | 50,000 |  |  |  |  |
| **8 Gy** | 5000 |  |  |  | 5000 | 12,500 |
|  | 8000 |  |  |  |  |  |
